# COVID-19 consequences on mental health: An African perspective

**DOI:** 10.4102/sajpsychiatry.v27i0.1611

**Published:** 2021-01-11

**Authors:** Matete R. Magamela, Tafadzwa Dzinamarira, Mbuzeleni Hlongwa

**Affiliations:** 1 Department of Public Health, School of Health Care Sciences, Sefako Makgatho Health Sciences University, Pretoria, South Africa; 2 Discipline of Public Health, School of Nursing and Public Health, University of KwaZulu-Natal, Durban, South Africa

## Background

Coronavirus disease 2019 (COVID-19) is a pandemic that has affected countries globally. At the time of writing, more than 21 million COVID-19 cases have been reported globally, including over 750 000 deaths. In Africa, over 1 million cases of COVID-19 have been reported, with over 25 000 deaths. Many countries have implemented various containment and mitigatory measures to slow the spread of the virus, including lockdowns, isolations and quarantines. However, the implementation of lockdowns, isolations and quarantines has introduced unintended alarming threats to mental health amongst populations. In this perspective, the authors discuss how COVID-19 lockdowns, isolations and quarantines have impacted mental health amongst populations.

The COVID-19 outbreak in 2019, caused by severe acute respiratory syndrome coronavirus 2 (SARS-CoV-2), is a pandemic affecting countries worldwide.^1^ At present, there is no vaccine or effective antiviral treatment for COVID-19, albeit numerous clinical trials are underway. Many countries have, appropriately, implemented lockdowns to reduce the rapid spread of COVID-19 to avoid overwhelming the healthcare systems. At the time of writing, the African continent had reported over a million cases of COVID-19, with over 25 000 succumbing to the deadly virus.^2^

## Coronavirus disease 2019 lockdowns, isolation and quarantine and consequences on mental health

Essential COVID-19 responses have been implemented to slow the spread of the virus, including lockdowns, isolations and quarantines. Quarantine is the process of separating and restricting the movement of people who have potentially been exposed to a contagious disease to reduce the risk of their infecting others.^3^ Lockdown is the process of limiting or reducing the movement and activities of people in the communities to reduce the rapid spread of COVID-19, and also adequately prepare healthcare systems to save lives. While the implementation of these measures is prudent to curb the rapid spread of COVID-19, it has left many people experiencing heightened feelings of uncertainty, unrest, loneliness, job losses, grief, no social interactions, gender-based violence, substance abuse and post-traumatic distress as a result of physical isolation from friends, family and community networks.^4^ The above-mentioned effects have been even more devastating for people diagnosed with COVID-19 and isolated. Research suggests that restrictive measures such as quarantine, isolation and social distancing have an impact on psychological well-being of people.^5^ These mental health challenges could have a lasting effect on the overall well-being of the population.^6^ In the African region, the prevalence of gender-based violence and substance abuse is high, and these are some of the major challenges that may arise following isolation and reduced social contact. Poverty and lack of resources remain one of the key issues likely to force people out of restrictions associated with COVID-19. For example, many people are unable to practice regular hand-washing because of poor access to water supply or sanitizers.

The African region has a shortage of mental health resources, medical professionals and infrastructure, while a massive disease burden continues to exist. This inevitably puts increased pressure on the already inadequate mental health resources in Africa. Even health care professionals are experiencing mental health problems because of the increased work demands, fatigue and unconducive working environment.^7^ Challenges related to mental health resources are likely to continue for longer periods and a range of mental disorders will continue to go undiagnosed, leading to further psychological harm.^8^

## Conclusion

In conclusion, while the current focus on fighting COVID-19 is critical at this moment, the ever-present mental and public health threat should not be ignored. The emerging mental health issues resulting from the COVID-19 pandemic may evolve into long-lasting health problems. Therefore, health measures should be implemented to address mental health issues related to COVID-19 lockdowns, isolations, gender-based violence and vulnerability amongst populations. Mental health interventions should be integrated into public health plans and emergency responses, and given the priority it deserves.
